# The Interaction of LFA-1 on Mononuclear Cells and ICAM-1 on Tubular Epithelial Cells Accelerates TGF-β1-Induced Renal Epithelial-Mesenchymal Transition

**DOI:** 10.1371/journal.pone.0023267

**Published:** 2011-08-05

**Authors:** Yoshiyuki Morishita, Minami Watanabe, Eiko Nakazawa, Kenichi Ishibashi, Eiji Kusano

**Affiliations:** 1 Division of Nephrology, Department of Medicine, Jichi Medical University, Tochigi, Japan; 2 Department of Medical Physiology, Meiji Pharmaceutical University, Tokyo, Japan; University of Sao Paulo – USP, Brazil

## Abstract

The epithelial-mesenchymal transition (EMT) of renal epithelial cells (RTECs) has pivotal roles in the development of renal fibrosis. Although the interaction of lymphocyte function-associated antigen 1 (LFA-1) on leukocytes and its ligand, intracellular adhesion molecule 1 (ICAM-1), plays essential roles in most inflammatory reactions, its pathogenetic role in the EMT of RTECs remains to be clarified. In the present study, we investigated the effect of the interaction of LFA-1 on peripheral blood mononuclear cells (PBMCs) and ICAM-1 on HK-2 cells after stimulation with TGF-β_1_ on the EMT of RTECs. ICAM-1 was highly expressed in HK-2 cells. After TGF-β_1_ stimulation, the chemokines CCL3 and CXCL12 increased on HK-2 cells. After co-culture of PBMCs and HK-2 cells pre-stimulated with TGF-β_1_ (0.1 ng/ml) (HK-2-TGF-β_1_ (0.1)), the expression of the active form of LFA-1 increased on PBMCs; however, total LFA-1 expression did not change. The expression of the active form of LFA-1 on PBMCs did not increase after co-culture with not CCL3 but CXCL12 knockdown HK-2-TGF-β_1_ (0.1). The expression of epithelial cell junction markers (E-cadherin and occludin) further decreased and that of mesenchymal markers (vimentin and fibronectin) further increased in HK-2-TGF-β_1_ (0.1) after co-culture with PBMCs for 24 hrs (HK-2-TGF-β_1_ (0.1)-PBMCs). The phosphorylation of ERK 1/2 but not smad2 and smad3 increased in HK-2-TGF-β_1_ (0.1)-PBMCs. The snail and slug signaling did not increase HK-2-TGF-β_1_ (0.1)-PBMCs. Although the migration and invasion of HK-2 cells induced full EMT by a high dose (10.0 ng/ml) and long-term (72–96 hrs) TGF-β_1_ stimulation increased, that of HK-2-TGF-β_1_ (0.1)-PBMCs did not increase. These results suggested that HK-2 cells stimulated with TGF-β_1_ induced conformational activation of LFA-1 on PBMCs by increased CXCL12. Then, the direct interaction of LFA-1 on PBMCs and ICAM-1 on HK-2 cells activated ERK1/2 signaling to accelerate the part of EMT of HK-2 cells induced by TGF-β_1._

## Introduction

Regardless of the underlying etiology, tubulointerstitial fibrosis is a common mechanism in the progression of chronic kidney disease (CKD) to end-stage renal disease [1], [2]. This progressive pathway involves interstitial infiltration by inflammatory mononuclear leukocytes [1], [3]. Integrin lymphocyte function-associated antigen 1 (LFA-1: α_L_β_2_ integrin) is the predominant integrin on leukocytes and an important molecule in firm adhesion and migration of leukocytes to inflammatory sites [4], [5]. LFA-1 also plays pivotal roles as a signal transduction molecule by binding its ligand, namely, intracellular adhesion molecule 1 (ICAM-1) [6], [7]. Normally, LFA-1 is expressed in a low-affinity state for its ligand and, thus, cells do not make unnecessary adhesive contacts while in circulation [8], [9]. The affinity of LFA-1 for ICAM-1 is mediated by a conformational change of LFA-1 and they play essential roles in most inflammatory reactions [8], [9]. ICAM-1 has been reported to be expressed on renal tubular epithelial cells (RTECs) and the expression of ICAM-1 on RTECs was found to be associated with the infiltration of leukocytes in CKD [10], [11]. An experimental animal study showed that ICAM-1 was promptly up-regulated after renal injury and leukocyte infiltration subsequently occurred [12]. Kelly et al. reported that anti-ICAM-1 mAb mitigated leukocyte infiltration in tubulointerstitial space in an ischemic renal injury animal model [13]. Although these results suggested that ICAM-1 on RTECs and LFA-1 on leukocytes have some roles in the progression of renal diseases, the pathogenetic roles of their direct interaction in renal fibrosis remain unclear.

Epithelial-mesenchymal transition (EMT) plays pivotal roles in organ fibrosis including that of kidney [14], [15]. It has been reported that a large proportion of the interstitial fibroblasts in fibrotic kidneys originate from proximal tubular cells [16]. Therefore, it is important to identify the molecules involved in the induction and progression of EMT of RTECs. TGF-β_1_ is up-regulated in the fibrotic kidney and is the main inducer of EMT of RTECs [17]–[18]. In the present study, we investigated the roles of the interaction of LFA-1 on peripheral blood mononuclear cells (PBMCs) and ICAM-1 on RTECs after stimulation of TGF-β_1_ on the EMT.

## Results

### ICAM-1 expression on HK-2 cells

**Figure 1:**
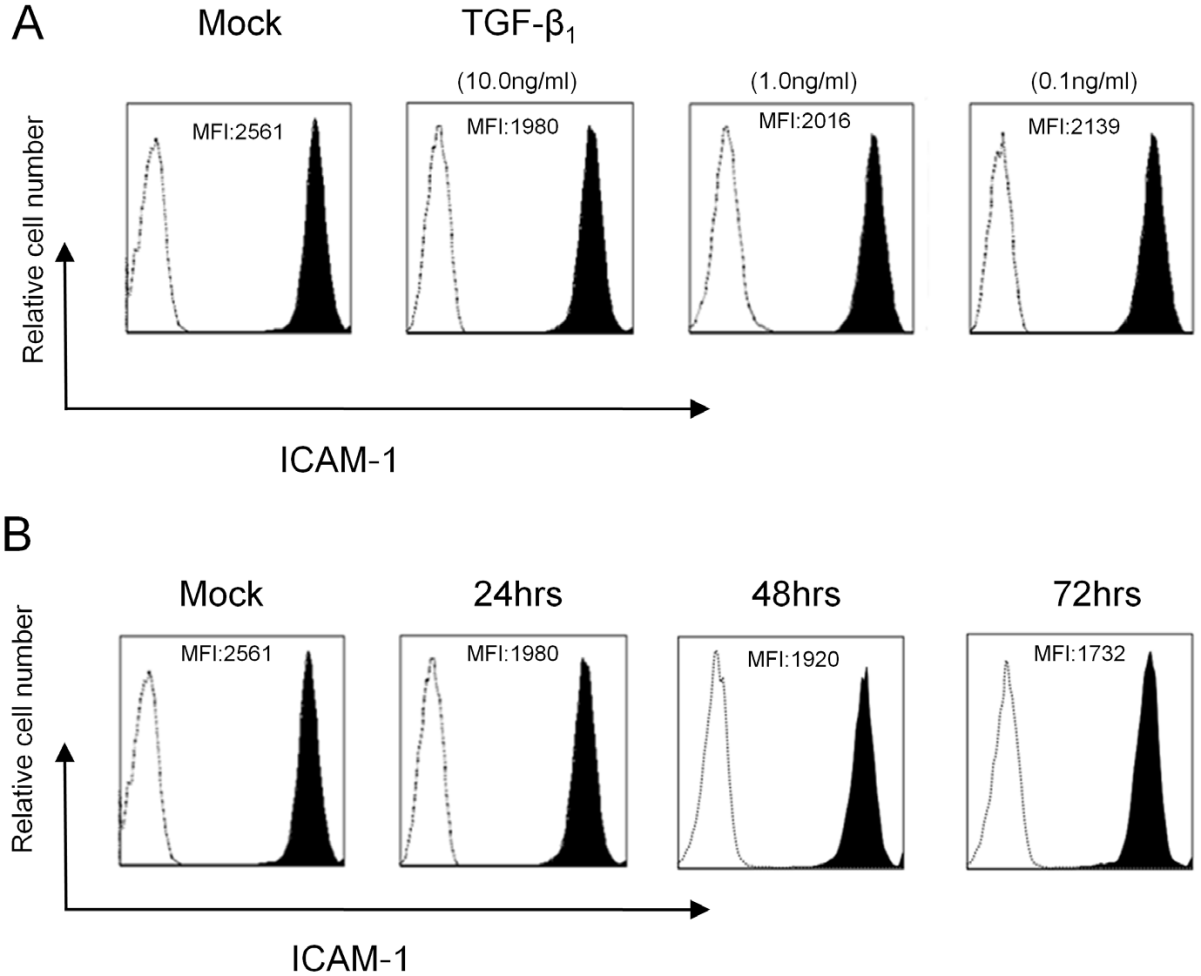
ICAM-1 expression on HK-2 cells. HK-2 cells (Mock) and HK-2 cells stimulated with TGF-β_1_ (10.0, 1.0 and 0.1 ng/ml) were stained with anti-ICAM-1 mAb and isotype control mAb (A). HK-2 cells (Mock) and HK-2 cells stimulated with TGF-β_1_ (10.0 ng/ml) were stained with anti-ICAM-1 mAb and isotype control mAb at 24 hrs, 48 hrs and 72 hrs after TGF-β_1_ stimulation (B). Cells were analyzed by flow cytometry. Closed histogram: ICAM-1-stained cells. Dotted line: isotype control stained cells. MFI: mean fluorescence intensity.

ICAM-1 was highly expressed on HK-2 cells. ICAM-1 expression decreased with TGF-β_1_ stimulation at concentrations of 10.0 ng/ml, 1.0 ng/ml and 0.1 ng/ml after 24 hrs in a dose-dependent manner (Figure 1A); its expression also showed a time-dependent decrease at 24 hrs, 48̀ hrs and 72 hrs after TGF-β_1_ (10.0 ng/ml) stimulation (Figure 1B). However, its expression was still maintained at a high level.

### TGF-β_1_ increased the expression of chemokines that mediate LFA-1 activation of PBMCs on HK-2 cells

**Figure 2:**
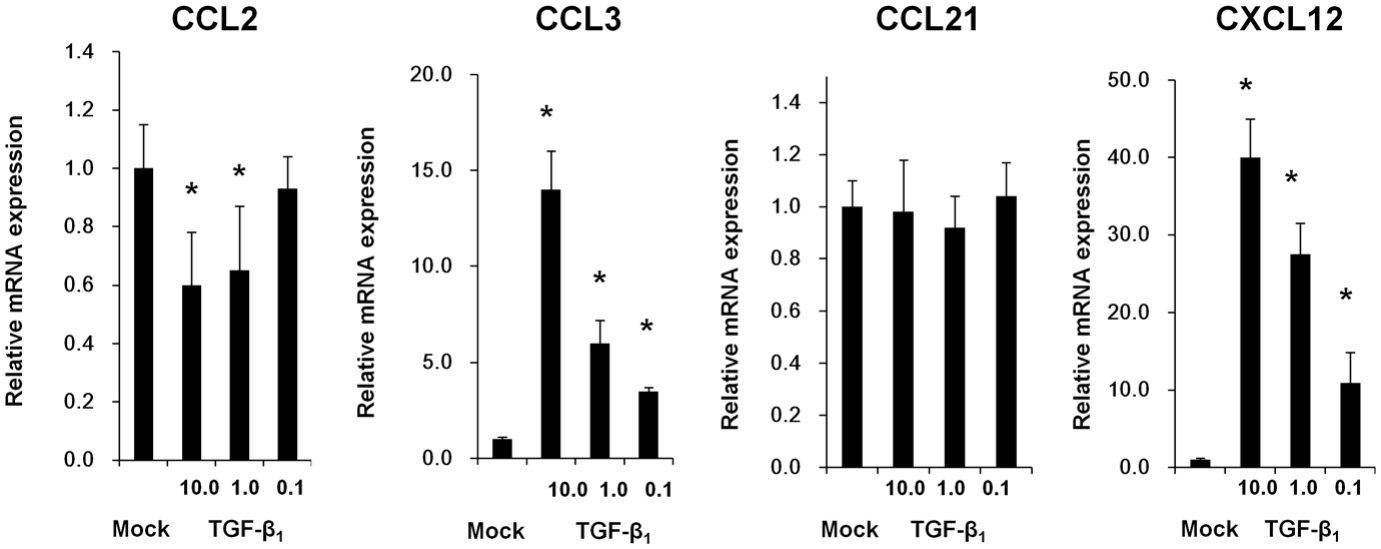
Effect of TGF-β_1_ on chemokine regulation of HK-2 cells. HK-2 cells were stimulated with TGF-β_1_ (10.0, 1.0 and 0.1 ng/ml) for 24 hrs. The expression changes of CCL3, CCL4, CCL17, CCL19, CCL20, CCL22 and CXCL12 were analyzed by real-time RT-PCR. CCL2 was down-regulated but CCL3 and CXCL12 were up-regulated on HK-2 cells by TGF-β_1_ stimulation in a dose-dependent manner. CCL21 did not change on HK-2 cells by TGF-β_1_ stimulation. Values are expressed as mean ± SEM of at least three experiments. *P<0.05.

The change of expression of chemokines that mediate LFA-1 activation on PBMCs, such as CCL2, CCL3, CCL4, CCL5, CCL17, CCL19, CCL20, CCL21, CCL22 and CXCL12, was investigated on HK-2 cells after stimulation of TGF-β_1_ (10.0 ng/ml, 1.0 ng/ml and 0.1 ng/ml). Although the expression of CCL2 decreased, the expression of CCL3 and CXCL12 increased on HK-2 cells after TGF-β_1_ stimulation at concentrations of 10.0 ng/ml, 1.0 ng/ml and 0.1 ng/ml for 24 hrs in a dose-dependent manner (Figure 2). The expression of CCL21 was not changed on HK-2 cells after TGF-β_1_ stimulation (Figure 2). CCL4, CCL5, CCL17, CCL19, CCL20 and CCL22 were not detected on HK-2 cells either before or after TGF-β_1_ stimulation (data not shown).

### HK-2 cells stimulated with TGF-β_1_ induced the conformational activation of LFA-1 on PBMCs

**Figure 3:**
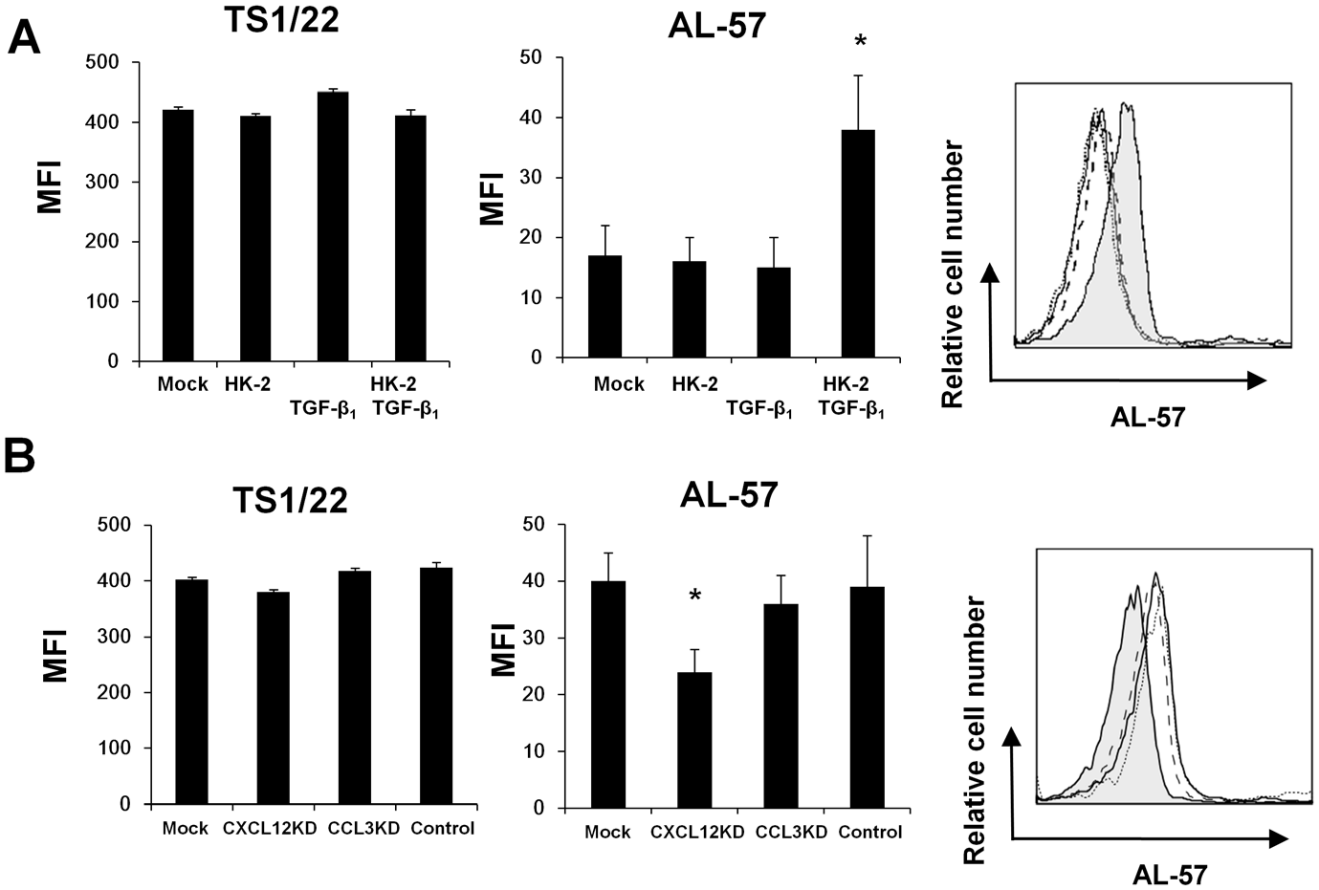
Effect of HK-2 cells on LFA-1 activation on PBMCs. PBMCs were grown alone (Mock), with HK-2 cells (HK-2), with TGF-β_1_ (0.1 ng/ml) (TGF-β_1_) or with HK-2 cells following TGF-β_1_ stimulation (HK-2-TGF-β_1_) for 30 minutes. Total LFA-1 on PBMCs and the active form of LFA-1 were stained with anti-LFA-1 mAb or the anti-active form of LFA-1 mAb. Closed histogram: AL57-stained PBMCs co-cultured with HK-2 cells following TGF-β_1_ (0.1 ng/ml) stimulation. Solid line: AL57-stained PBMCs (Mock). Dashed line: AL57-stained PBMCs co-cultured with HK-2 cells. Dotted line: AL57-stained PBMCs stimulated with TGF-β_1_ (0.1 ng/ml) alone. The graphs represent relative changes of total LFA-1 (TS1/22) and the active form of LFA-1 (AL-57) on HK-2 cells as mean ± SEM of at least three experiments. *P<0.05. MFI: mean fluorescence intensity (A). PBMCs were grown with non-treated HK-2 cells (Mock), CXCL12 knockdown HK-2 cells (CXCL12KD), CCL3 knockdown HK-2 cells (HCCL3KD) or non-targeted-siRNA-transfected HK-2 cells (Control) following TGF-β_1_ (0.1 ng/ml) stimulation for 30 minutes. Total LFA-1 on PBMCs and the active form of LFA-1 were stained with anti-LFA-1 mAb or the anti-active form of LFA-1 mAb. Cells were analyzed by flow cytometry. Closed histogram: AL57-stained PBMCs co-cultured with CXCL12 KD following TGF-β_1_ (0.1 ng/ml) stimulation. Solid line: AL57-stained PBMCs co-cultured with Mock following TGF-β_1_ (0.1 ng/ml) stimulation. Dashed line: AL57-stained PBMCs co-cultured with CCL3KD following TGF-β_1_ (0.1 ng/ml) stimulation. Dotted line: AL57-stained PBMCs co-cultured with Control following TGF-β_1_ (0.1 ng/ml) stimulation. Values are expressed as mean ± SEM of at least three experiments. *P<0.05. MFI: mean fluorescence intensity (B).

We investigated the conformational activation change of LFA-1 on PBMCs by HK-2 cells after stimulation with TGF-β_1_ (0.1 ng/ml). We employed flow cytometry using anti-LFA-1 mAb (clone: TS1/22) to detect total LFA-1 on PBMCs and anti-active form LFA-1 mAb (clone: AL-57) that reacted only with the active form of LFA-1 but not with the resting form of LFA-1 to detect conformational activation of LFA-1 on PBMCs. After co-culture with HK-2 cells stimulated by TGF-β_1_ (0.1 ng/ml) for 30 minutes, AL57-positive cells were significantly increased , whereas TS1/22-positive cells were not changed on PBMCs. AL57-positive cells on PBMCs did not change by co-culture with HK-2 cells without TGF-β_1_ and TGF-β_1_ alone (Figure 3A). Next, we investigated the effect of CCL3 and CXCL12 of HK-2 cells on the LFA-1 activation on HK-2 cells. CXCL12 and CCL3 on HK-2 cells were knocked down by transfection of CXCL12-siRNA or CCL3-siRNA, respectively. Non-treated (Mock) HK-2 cells and non-targeted-siRNA-transfected (Control) HK-2 cells served as controls. CXCL12 knockdown (CXCL12 KD) HK-2 cells did not increase CXCL12 expression regardless of TGF-β_1_ stimulation. CCL3 knockdown (HK-2-CCL3 KD) HK-2 cells did not increase CCL3 expression regardless of TGF-β_1_ stimulation (Figure S1). PBMCs were co-cultured with CXCL12 KD, CCL3 KD, Control or Mock HK-2 cells after stimulation with TGF-β_1_ (0.1 ng/ml) for 30 minutes. Significantly less AL57-positive cells were detected among PBMCs after co-culture with CXCL12KD HK-2 cells than among CCL3KD, Control and Mock HK-2 cells (Figure 3B).

### Interaction of LFA-1 on PBMCs and ICAM-1 on HK-2 cells accelerated TGF-β_1_-induced EMT of HK-2 cells

**Figure 4:**
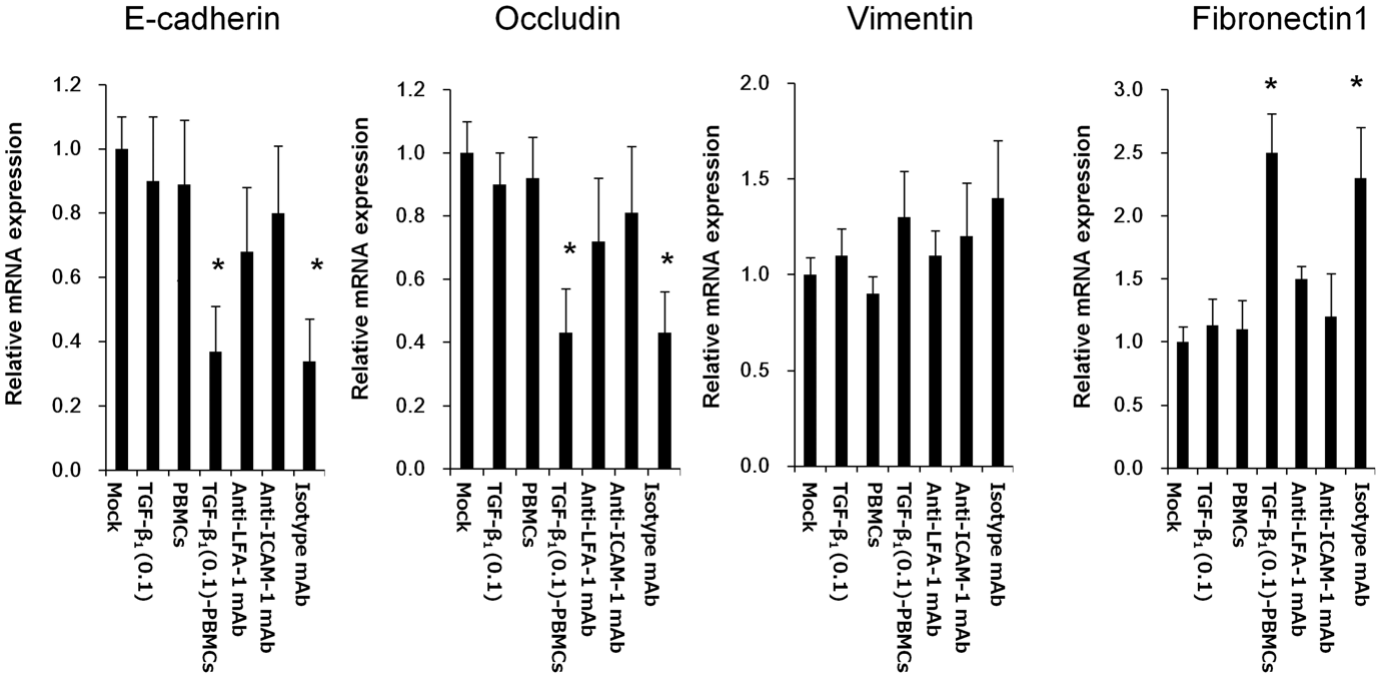
Effect of PBMCs on EMT markers on HK-2 cells. HK cells were co-cultured with PBMCs for 24 hrs following TGF-β_1_ (0.1 ng/ml) stimulation for 24 hrs. Then, the changes of the epithelial markers (E-cadherin and occludin) and the mesenchymal markers (vimentin and fibronectin 1) on HK-2 cells (HK-2-TGF-β_1_ (0.1)-PBMCs) were assessed by real-time RT-PCR. For blocking the interaction of LFA-1 and ICAM-1, before co-culture, PBMCs were pre-incubated with anti-LFA-1 mAb for 20 minutes (Anti-LFA-1-mAb) or HK-2 cells were pre-incubated with anti-ICAM-1 mAb for 20 minutes (Anti-ICAM-1mAb) or isotype control mAb for 20 minutes (Isotype mAb). The changes of epithelial cell junction markers and mesenchymal markers on HK-2 cells without co-culture with PBMCs following TGF-β_1_ (0.1 ng/ml) stimulation for 24 hrs (HK-2-TGF-β_1_ (0.1)) and HK-2 cells without stimulation of TGF-β_1_ co-cultured with PBMCs (HK-2-PBMCs) were investigated as controls. Values are expressed as mean ± SEM of at least three experiments. *P<0.05.

**Figure 5:**
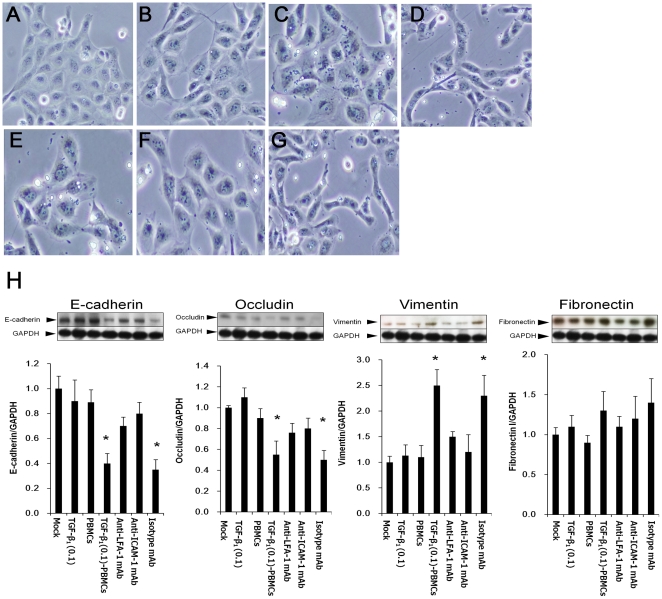
Assessment of the effect of PBMCs on the morphology and EMT markers of HK-2 cells. Non-treated HK-2 cells (A). HK-2 cells grown for 24 hrs following TGF-β_1_ (0.1 ng/ml) stimulation for 24 hrs (B). HK-2 cells co-cultured with PBMCs for 24 hrs without TGF-β_1_ stimulation (C). HK-2 cells co-cultured with PBMCs for 24 hrs following TGF-β_1_ (0.1 ng/ml) stimulation (D). HK-2 cells co-cultured with PBMCs with pre-incubated anti-LFA-1 mAb following TGF-β_1_ (0.1 ng/ml) stimulation for 24 hrs (E). HK-2 cells pre-incubated with anti-ICAM-1 mAb co-cultured with PBMCs following TGF-β_1_ (0.1 ng/ml) stimulation for 24 hrs (F). HK-2 cells pre-incubated with isotype control mAb co-cultured with PBMCs following TGF-β_1_ (0.1 ng/ml) stimulation for 24 hrs (G). Magnification 100×. HK cells were co-cultured with PBMCs for 24 hrs following TGF-β_1_ (0.1 ng/ml) stimulation for 24 hrs. Then, the changes of the epithelial markers (E-cadherin and occludin) and the mesenchymal markers (vimentin and fibronectin 1) on HK-2 cells (HK-2-TGF-β_1_ (0.1)-PBMCs) were assessed by western blotting analysis (H). For blocking the interaction of LFA-1 and ICAM-1, before co-culture, PBMCs were pre-incubated with anti-LFA-1 mAb for 20 minutes (Anti-LFA-1 mAb ) or HK-2 cells were pre-incubated with anti-ICAM-1 for 20 minutes (Anti-ICAM-1 mAb) or isotype control mAb for 20 minutes (Isotype mAb). HK-2 cells without co-culture with PBMCs following TGF-β_1_ (0.1 ng/ml) stimulation for 24 hrs (TGF-β_1_ (0.1)) and HK-2 cells without stimulation of TGF-β_1_ co-cultured with PBMCs (PBMCs) were investigated as control. One representative immunoblot is presented at the top of the graph. Values are expressed as mean ± SEM of at least three experiments. *P<0.05.

We investigated the impact of the interaction of LFA-1 on PBMCs and ICAM-1 on HK-2 cells on the EMT of HK-2 cells. HK-2 cells after stimulation of TGF-β_1_ (0.1 ng/ml) for 24 hrs were co-cultured with PBMCs for a further 24 hrs (HK-2-TGF-β_1_ (0.1)-PBMCs). Then, the changes of epithelial cell junction markers and mesenchymal markers on HK-2-TGF-β_1_ (0.1)-PBMCs were investigated by real-time RT-PCR and western blotting analysis. HK-2 cells after stimulation of TGF-β_1_ (0.1 ng/ml) without co-culture with PBMCs (HK-2-TGF-β_1_ (0.1)) and HK-2 cells without stimulation of TGF-β_1_ co-cultured with PBMCs (HK-2-PBMCs) were investigated as control experiments. The results of real-time RT-PCR showed that the expression of epithelial cell junction markers (E-cadherin and occludin) further significantly decreased and that of mesenchymal markers (vimentin and fibronectin 1) further significantly increased in HK-2-TGF-β_1_ (0.1)-PBMCs compared with those of controls. These EMT marker changes on HK-2-TGF-β_1_ (0.1)-PBMCs were blocked by anti-LFA-1 mAb or anti-ICAM-1 mAb, whereas they were not blocked by isotype control mAb (Figure 4). Phase contrast microscopy analysis showed that HK-2-TGF-β_1_ (0.1)-PBMCs changed more, morphologically, from a cobblestone-like monolayer to spindle-shaped fibroblast-like cells, than the controls. These morphological changes of HK-2-TGF-β_1_ (0.1)-PBMCs were mitigated by anti-LFA-1 mAb or anti-ICAM-1 mAb, whereas they were not mitigated by isotype control mAb (Figure 5 A–G). Western blot analysis showed that the expression of epithelial cell junction markers (E-cadherin and occludin) further significantly decreased and that of mesenchymal markers (vimentin and fibronectin) further significantly increased in HK-2-TGF-β_1_ (0.1)-PBMCs compared with those of controls. These EMT marker changes on HK-2-TGF-β_1_ (0.1)-PBMCs were blocked by anti-LFA-1 mAb or anti-ICAM-1 mAb, whereas they were not blocked by isotype control mAb (Figure 5 H).

### The interaction of HK-2 cells pre-stimulated with TGF-β_1_ and PBMCs up-regulated TGF-β_1_ level in co-culture media

**Figure 6:**
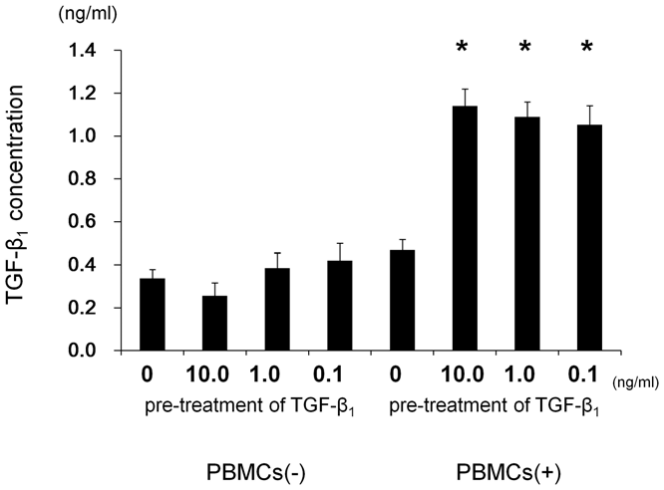
The effect of the co-culture of HK-2 cells pre-stimulated with TGF-β_1_ and PBMCs for TGF-β_1_ level in culture media. HK-2 cells without TGF-β_1_ stimulation and PBMCs were co-cultured in lower plate and HK-2 cells with pre-stimulated TGF-β_1_ (10.0 ng/ml, 1.0 ng/ml and 0.1 ng/ml) were cultured in upper insert in transwell plate. After 24 hrs of incubation, TGF-β_1_ concentrations in the lower well media were determined by TGF-β_1_ ELISA. Values are expressed as mean ± SEM of at least three experiments. *P<0.05.

To investigate whether pre-stimulation of HK-2 cells by TGF-β_1_ contributes to EMT or just induces the CXCL12 that activates LFA-1 on PBMCs, we employed a transwell co-culture system as described above. HK-2 cells without TGF-β_1_ stimulation and PBMCs were co-cultured in a lower plate and HK-2 cells pre-stimulated by TGF-β_1_ (10.0 ng/ml, 1.0 ng/ml and 0.1 ng/ml) were cultured in an upper insert. After 24 hrs of incubation, TGF-β_1_ concentrations in the media in the lower wells were determined by TGF-β_1_ ELISA. The concentrations of TGF-β_1_ in the co-culture media of HK-2 cells and PBMCs in the lower wells were about threefold higher than those of HK-2 cells alone (Fig. 6).

### The interaction of LFA-1 on PBMCs and ICAM-1 on HK-2 cells activated ERK 1/2 signaling on HK-2 cells

**Figure 7:**
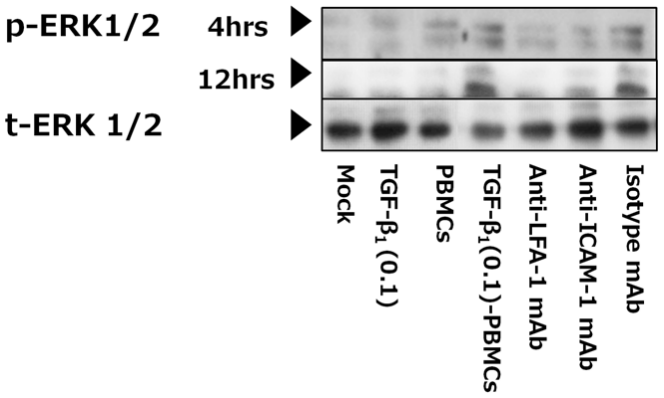
The effect of PBMCs on the activation of extracellular regulated kinase 1 and 2 (ERK1/2) of HK-2 cells. HK cells were co-cultured with PBMCs for 24 hrs following TGF-β_1_ (0.1 ng/ml) stimulation for 24 hrs (TGF-β_1_ (0.1)-PBMCs). After 4 and 12 hrs of co-culture with PBMCs, aliquots of HK-2 cell lysate were subjected to western blot analysis. For blocking the interaction of LFA-1 and ICAM-1, before co-culture, PBMCs were pre-incubated with anti-LFA-1 mAb for 20 minutes (Anti-LFA-1 mAbs) and HK-2 cells were pre-incubated with anti-ICAM-1 mAb for 20 minutes (Anti-ICAM-1 mAbs) or isotype control mAb for 20 minutes (Isotype mAb). HK-2 cells without co-culture with PBMCs following TGF-β_1_ (0.1 ng/ml) stimulation for 24 hrs (TGF-β_1_(0.1)) and HK-2 cells without stimulation of TGF-β_1_ co-cultured with PBMCs (PBMCs) were investigated as controls. One representative immunoblot is presented at the top of the graph.

We investigated the phosphorylation of smad2, smad3 and ERK1/2 in HK-2-TGF-β_1_ (0.1)-PBMCs to investigate the signaling pathways that might be involved in the acceleration of TGF-β_1_-induced EMT on HK-2 cells by PBMCs. The phosphorylation of smad2 and smad3 was not detected (data not shown); however, the phosphorylation of ERK1/2 was detected in HK-2-TGF-β_1_ (0.1)-PBMCs at 4 hrs and 12 hrs after co-culture by western blot analysis (Figure 7). The phosphorylation of ERK1/2 was blocked by anti-LFA-1 mAb (clone: TS1/22; 10 µg/ml) or anti-ICAM-1 mAb (clone: 6.5B5; 10 µg/ml), whereas it was not blocked by isotype control mAb (Figure 7).

### The interaction of PBMCs and HK-2 cells pre-stimulated with TGF-β_1_ contributed to the part of EMT of HK-2 cells

**Figure 8:**
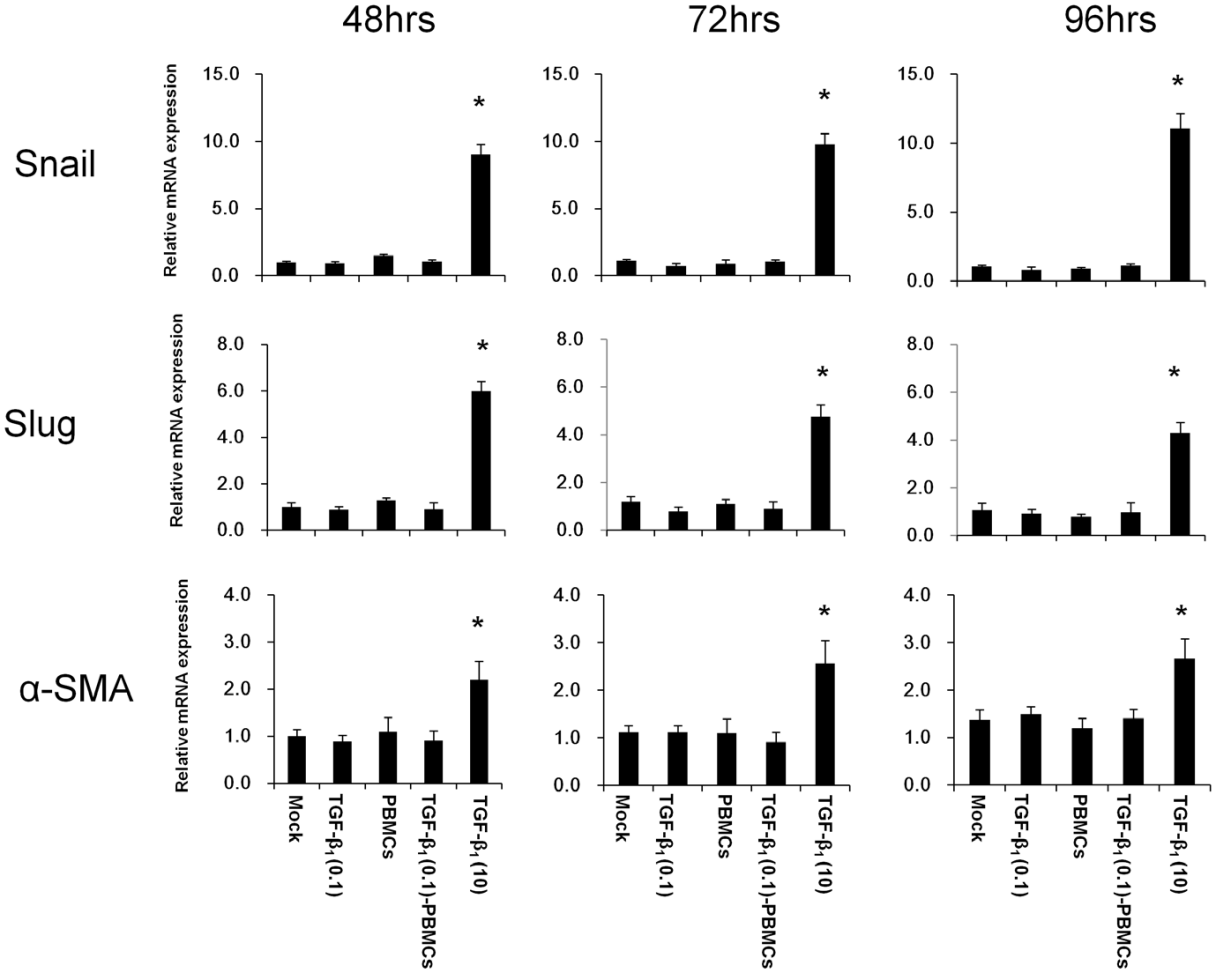
The effect of interaction of HK-2 cells pre-stimulated with TGF-β_1_ and PBMCs for snail and slug signaling and α-smooth muscle actin expression on HK-2 cells. The expressions of snail and slug signaling and α-smooth muscle actin (α-SMA) in the HK-2 cells pre-stimulated with TGF-β_1_ (0.1 ng/ml) followed by co-culture with PBMCs (TGF-β_1_ (0.1)-PBMC) for 48 hrs, 72 hrs and 96 hrs were determined by real-time RT-PCR. Non-treated HK-2 cells (Mock), HK-2 cells treated with TGF-β_1_ (0.1 ng/ml) (TGF-β_1_ (0.1)), HK-2 cells without TGF-β_1_ treatment with co-cultured with PBMCs (PBMCs) and HK-2 cells treated with TGF-β_1_ at 10 ng/ml (TGF-β_1_ (10)) served as controls. Values are expressed as mean ± SEM of at least three experiments. *P<0.05.

**Figure 9:**
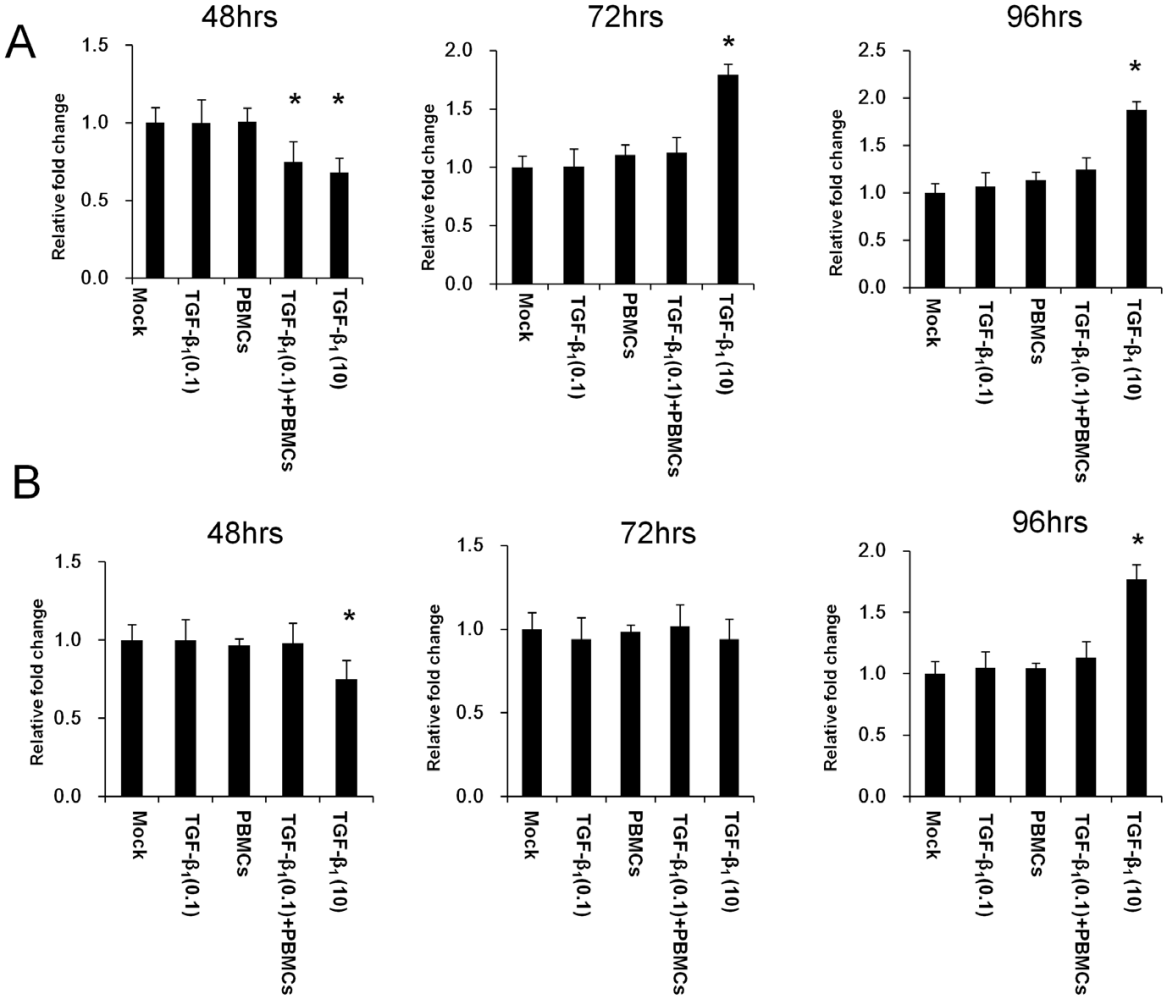
The effect of interaction of HK-2 cells pre-stimulated with TGF-β_1_ and PBMCs for the migration and invasion of HK-2 cells. The migration (A) and invasion (B) of HK-2 cells pre-stimulated with TGF-β_1_ (0.1 ng/ml) for 24 hrs followed by co-culture with PBMCs (TGF-β_1_ (0.1)-PBMCs) for 48 hrs, 72 hrs and 96 hrs were investigated by transwell assay. Non-treated HK-2 cells (Mock), HK-2 cells stimulated with TGF-β_1_ (0.1 ng/ml) (TGF-β_1_ (0.1)), HK-2 cells without TGF-β_1_ stimulation co-cultured with PBMCs, (PBMCs) and HK-2 cells stimulated with TGF-β_1_ at 10 ng/ml (TGF-β_1_ (10)) served as controls. Values are expressed as mean ± SEM of at least three experiments. *P<0.05.

We compared the role of the interaction of PBMCs and HK-2 cells pre-stimulated with TGF-β_1_ and that of TGF-β_1_ for EMT of HK-2 cells. The expression of snail and slug signaling and the expression of α-SMA in HK-2-TGF-β_1_ (0.1)-PBMC were investigated by real-time RT-PCR at 48 hrs, 72 hrs and 96 hrs after co-culture. HK-2 cells treated with TGF-β_1_ (10.0 ng/ml) (HK-2-TGF-β_1_ (10)) served as controls of TGF-β_1_-induced full EMT of HK-2 cells. Mock, HK-2-TGF-β_1_ (0.1) and HK-2-PBMCs were investigated as control experiments. Although snail and slug signaling and a-SMA expression significantly increased in HK-2-TGF-β_1_ (10) at 48 hrs, 72 hrs and 96 hrs, they did not increase in HK-2-TGF-β_1_ (0.1)-PBMCs, HK-2-TGF-β_1_ (0.1) and HK-2-PBMCs compared with those of Mock (Figure 8). Furthermore, we investigated the migration and invasion of HK-2-TGF-β_1_ (0.1)-PBMC. Although the migration of HK-2-TGF-β_1_ (10) significantly increased at 72 hrs and 96 hrs with a transient decrease at 48 hrs, that of HK-2-TGF-β_1_ (0.1)-PBMCs did not change compared with those of Mock, HK-2-TGF-β_1_ (0.1) and HK-2-PBMCs at 72 hrs and 96 hrs with a transient decrease at 48 hrs (Figure 9A). Although the invasion of HK-2-TGF-β_1_ (10) increased at 96 hrs with a transient decrease at 48 hrs, that of HK-2-TGF-β_1_ (0.1)-PBMC did not change compared with those of Mock, HK-2-TGF-β_1_ (0.1) and HK-2-PBMCs at 48 hrs, 72 hrs and 96 hrs (Figure 9B).

## Discussion

The present study showed that the TGF-β_1_-induced EMT on HK-2 cells was accelerated by PBMCs. The decrease of expression level of epithelial cell junction markers and increase of expression level of mesenchymal markers on HK-2 cells after stimulation of TGF-β_1_ were accelerated by co-culture with PBMCs. These EMT marker changes on HK-2 cells were mitigated by anti-LFA-1 mAb or anti-ICAM-1 mAb. These results demonstrated that the direct interaction of LFA-1 on PBMCs and ICAM-1 on HK-2 cells contributed to accelerate TGF-β_1_-induced EMT on HK-2 cells. HK-2 cells stimulated by TGF-β_1_ induced conformational activation of LFA-1 on PBMCs; however, HK-2 cells without stimulation by TGF-β_1_ or TGF-β_1_ alone did not induce conformational activation of LFA-1 on PBMCs. The locally secreted chemokines can trigger integrin-dependent adhesion of circulating leukocytes that leads to infiltration [19], [20], [21], [22]. Several studies have characterized the expression pattern of chemokines in animal models of tubulointerstitial disease [23], [24], [25]. They demonstrated that various chemokines such as CCL2, CCL3, CCL4 and CCL5 are expressed only in the diseased compartment of the kidney and renal chemokine expression has been found to correlate with the leukocyte accumulation area and renal damage [23], [24], [25], [26]. On the basis of these findings, we investigated the regulation of the chemokines that may mediate LFA-1 activation of PBMCs on HK-2 cells by the stimulation of TGF-β_1_. CCL2 decreased on HK-2 cells by stimulation of TGF-β_1._ This result was consistent with a previous study [27]. CCL3 and CXCL12 increased on HK-2 cells by TGF-β_1_ stimulation. CCL21 did not change on HK-2 cells by TGF-β_1_ stimulation. CCL3, CCL4, CCL17, CCL19, CCL20 and CCL22 were not detected on HK-2 cells either before or after TGF-β_1_ stimulation. CCL3 and CXCL12 were reported to activate LFA-1 on both monocytes and lymphocytes [28], [29], [30], [31], [32]. In the present study, not CCL3 but CXCL 12 contributed to induction of the conformational activation of LFA-1 on PBMCs, because less AL57-positive cells were detected among PBMCs after co-culture with CXCL12 knockdown HK cells compared with those among Mock, CCL3 knockdown and control knockdown HK-2 cells pre-stimulated with TGF-β_1._ Furthermore, the EMT marker changes of HK-2 cells pre-stimulated with TGF-β_1_ and morphological changes upon co-culture with PBMC were inhibited by CXCL12 knockdown on HK-2 cells (Figures S2 and S3). These results suggested that CXCL12 was a key chemokine for EMT of RTECs that induced TGF-β_1_ accelerated by PBMCs.

Since TGF-β_1_ is up-regulated in the fibrotic kidney and is the main inducer of EMT in RTECs [17], [18], we determined TGF-β_1_concentration in the co-culture media of HK-2 cells and PBMCs with non-direct contacted culture with HK-2 cells pre-stimulated by TGF-β_1_ using transwell plate to investigate whether pre-stimulation of HK-2 cells by TGF-β_1_ contributes to EMT on RTECs or just induces the CXCL12 that activates LFA-1 on PBMCs. TGF-β_1_ concentrations of the co-culture media of HK-2 cells and PBMCs were about threefold up-regulated compared with those of HK-2 cells without PBMCs. These results suggested that the pre-stimulation of HK-2 cells by TGF-β_1_ contributes to increase not only chemokine production but also up-regulation of TGF-β_1_ that induced EMT in RTECs upon the interaction of RTECs and PBMCs. We conducted real-time RT-PCR to detect the change of the *TGF-β_1_ mRNA* in the HK-2 cells that underwent EMT and PBMCs in the lower plate separately at several time points after co-culture; however, we could not detect up-regulation of *TGF-β_1_ mRNA* in both these HK-2 cells and PBMCs (data not shown). Although further study will be needed to investigate the regulation of TGF-β_1_ on the interaction of LFA-1 on PBMCs and ICAM-1 on RTECs, the up-regulation of TGF-β_1_ concentrations of the co-culture media of HK-2 cells and PBMCs also suggested the possibility of autocrine and self-sustained activation of TGF-β_1_ on RTECs in the presence of PBMCs.

In the EMT of RTECs, smad2 and smad3 were mainly mediated by TGF-β_1_ [33] . In the present study, the phosphorylation of smad2 and smad3 was not detected in HK-2 cells by the stimulation of TGF-β_1_ for 24 hrs before and after co-culture with PBMCs. This lack of phosphorylation of smad2 and smad3 is considered to be due to the low concentration of TGF-β_1_ (0.1 ng/ml) because phosphorylation of smad2 and smad3 on HK-2 cells was detected after stimulation with higher concentrations of TGF-β_1_ (10.0 ng/ml) for 24 hrs (data not shown). Xie et al. reported that the activation of ERK pathway was required for TGF-β_1_-induced EMT of RTECs [34]. Schramek et al. reported that long-term ERK1/2 activation was an important mechanism involved in the EMT of HK-2 cells [35], [36]. In addition to the role of ICAM-1 as an adhesion molecule, ICAM-1 also has roles as a signal transduction molecule [37], [38], [39]. With the interaction of LFA-1 with ICAM-1 and the formation of ICAM-1 multimers within the cell membrane, signal transduction is initiated [40], [41]. ICAM-1 with associated proteins such as Grb2, SOS and Shc can lead to downstream activation of the mitogen-activated protein (MAP) kinase pathway, including ERK1/2 [38], [42]. The ERK1/2 proteins were most commonly activated through ICAM-1 ligation [38], [40]. In the present study, phosphorylation of ERK1/2 was detected on HK-2 cells after stimulation of TGF-β_1_ co-cultured with PBMCs. This phosphorylation of ERK1/2 was blocked by anti-LFA-1 mAb or anti-ICAM-1 mAb. These results suggested that ERK 1/2 may be activated through ICAM-1 on HK-2 cells by the interaction of ICAM-1 on HK-2 cells and LFA-1 on PBMCs. LFA-1 also acts as a signal transduction molecule when it binds ligands [7]. Although ICAM-1 expression still showed a high level in HK-2 cells by TGF-β_1_ stimulation, its suppression by TGF-β_1_ might be an inhibition factor for EMT of HK-2 cells by the interaction of ICAM-1 of HK-2 cells and LFA-1 of PBMCs. Further study will be needed to investigate the mechanism and significance of the regulation of ICAM-1 expression, the signaling pathway that affects EMT of HK-2 cells on PBMCs by the interaction of LFA-1 on PBMCs and ICAM-1 on HK-2 cells.

Next, we characterized the EMT of HK-2-TGF-β_1_ (0.1)-PBMCs compared with full EMT of HK-2 induced by TGF-β_1_ (HK-2-TGF-β_1_ (10)). Although snail and slug signaling and a-SMA expression significantly increased in HK-2-TGF-β_1_ (10), they did not increase in HK-2-TGF-β_1_ (0.1)-PBMCs at 48 hrs, 72 hrs and 96 hrs, the same as HK-2-PBMCs and HK-2-TGF-β_1_ (0.1). Furthermore, the migration of HK-2-TGF-β_1_ (10) significantly increased at 72 hrs and 96 hrs with a transient decrease at 48 hrs; however, that of HK-2-TGF-β_1_ (0.1)-PBMCs did not change compared with those of Mock, HK-2-TGF-β_1_ (0.1) and HK-2-PBMCs at 72 hrs and 96 hrs with a transient decrease at 48 hrs. Although the invasion of HK-2-TGF-β_1_ (10) also increased at 96 hrs with a transient decrease at 48 hrs, that of HK-2-TGF-β_1_ (0.1)-PBMC did not change compared with those of Mock, HK-2-TGF-β_1_ (0.1) and HK-2-PBMCs at 48 hrs, 72 hrs and 96 hrs. These results suggested that TGF-β_1_ (0.1) is not sufficient to induce EMT of RTECs and the interaction of LFA-1 of PBMCs and ICAM-1 of RTECs pre-stimulated with TGF-β_1_ (0.1) contributed to the early part of EMT of RTECs. Taking these findings together, the low level of TGF-β_1_ that could not induce EMT of RTECs by itself may contribute to EMT of RTECs by inducing the interaction of ICAM-1 of RTECs and LFA-1 on PBMCs, leading to activation of the signaling through ICAM-1 and self-sustained activation of TGF-β_1_ on RTECs_._

The extent to which EMT contributes to renal fibrosis remains to be clarified; however, evidence for the role of EMT in renal fibrosis in various renal diseases is emerging. A large proportion of the interstitial fibroblasts in fibrotic kidneys have been reported to originate from proximal tubular cells in an animal model [16]. Clinical studies utilizing human kidney biopsies also suggest that EMT plays a role in the pathogenesis of renal fibrosis [43], [44], [45]. Furthermore, EMT of RTECs was well correlated with declining renal function [43], [45]. The renal fibrosis also involved interstitial infiltration of inflammatory mononuclear leukocytes [3]. Thus, our finding suggested that inflammatory mononuclear leukocytes contributed to renal fibrosis by accelerating EMT of RTECs.

In conclusion, HK-2 cells stimulated with TGF-β_1_ induced activation of LFA-1 on PBMCs by increased CXCL12. The direct interaction of LFA-1 on PBMCs and ICAM-1 on HK-2 cells activated ERK1/2 signaling to accelerate the part of EMT of HK-2 cells induced by TGF-β_1._ These results suggested that drugs that can block the interaction of LFA-1 and ICAM-1 could be an option to treat renal fibrosis.

## Materials and Methods

### Cell culture

HK-2 cells (human renal proximal tubular epithelial cells immortalized by transduction with human papilloma virus 16 E6/E7 genes) were purchased from American Type Culture Collection (Manassas, VA). HK-2 cells were cultured in RPMI 1640 (Sigma-Aldrich, St. Louis, MO) supplemented with 10% fetal bovine serum (FBS) (Invitrogen, Grand Island, NY) and 1% penicillin and streptomycin (Invitrogen). PBMCs were obtained from three healthy donors, after obtaining informed consent, using Ficoll-gradient sedimentation. PBMCs were cultured with RPMI 1640 (Sigma-Aldrich) supplemented with 10% FBS (Invitrogen) and 1% penicillin and streptomycin (Invitrogen).

### Treatments

The sub-confluent cultures of HK-2 cells in 6-well plate (Falcon, Franklin Lake, NJ) were treated with recombinant human TGF-β_1_ (R&D Systems, Minneapolis, MN, USA) at concentrations of 10.0, 1.0 and 0.1 ng/ml for 24 hrs. Next, the cells were washed three times with phosphate-buffered saline (PBS). Then, PBMCs (2×10^5^) were added to each well for co-culture for 24 hrs. For blocking the interaction of LFA-1 on PBMCs and ICAM-1 on HK-2 cells, HK-2 cells were pre-incubated for 20 minutes at 37°C with 20 µg/ml anti-ICAM-1 monoclonal antibody (mAb) (clone: 6.5B5, Santa Cruz Biotechnology Inc., Santa Cruz, CA) or naïve PBMCs were pre-incubated for 20 minutes at 37°C with µg/ml anti-LFA-1 mAb (clone TS1/22, kindly provided by Dr. M. Shimaoka, IDI Harvard Medical School, Boston) before co-culture. Pre-incubation with HK-2 and PBMCs with mouse IgG_1_ isotype control mAb (clone MOPC-31C, BD, San Jose, CA) was used for control experiments.

### Real-time RT-PCR

Total RNA from cells was isolated with RNeasy total RNA isolation kit (Qiagen, Valencia, CA). The isolated 1 µg of total RNA was reverse-transcribed using Superscript III first-strand synthesis system (Invitrogen) according to the manufacturer's protocol. Real-time RT-PCR was performed using SYBR Green ER qPCR super mix (Invitrogen) and an Applied Biosystems Step One Plus Real-Time PCR system (Applied Biosystems, Carlsbad, CA). All reactions were carried out in a 20 µl reaction volume in triplicate. Primers for human GAPDH, E-cadherin, occludin, vimentin, fibronectin 1, α-smooth muscle actin, snail, slug, CCL2, CCL3, CCL4, CCL5, CCL17, CCL19, CCL20, CCL21, CCL22 and CXCL12 were as previously described [46,47,48,49,50,51,52,53, Lin, 2009 #23, Hotz, 2007 #125].

### Flow cytometry

Cells were stained with specific mAbs and analyzed using a BD-LSR flow cytometer (BD). The antibodies used were anti-ICAM-1 mAb (clone 15.2, Abcam, Cambridge, MA), anti-LFA-1 mAb, anti-active form LFA-1 mAb that reacts only with the active conformational form of LFA-1 but does not react with the non-active conformational form (clone AK-57, kindly provided by Dr. M. Shimaoka, IDI, Harvard Medical School, Boston) [54], mouse IgG_1_ isotype control mAb (clone MOPC-31C, BD), PE-conjugated goat anti-mouse immunoglobulin-specific antibody (BD) and PE-conjugated F(ab')_2_ fragment anti-human IgG (H+L) (Jackson Immuno Research, West Grove, PA).

### Western blot analysis

For western blotting, cells were extracted with lysis buffer [20 mM Tris-HCl (pH 7.4), 1% Triton, 10% glycerol and 0.1 mM phenylmethylsulfonyl fluoride] and phosphatase inhibitors (2 mM sodium fluoride, 2 mM sodium pyrophosphate, 1 mM sodium orthovanadate). Equal amounts of protein samples (40 g) were electrophoresed on NuPAGE sodium dodecyl sulphate–polyacrylamide gels (Invitrogen), and then transferred onto Immobilon-P membranes (Millipore Corporation, Bedford). After nonspecific protein binding was blocked with skim milk and bovine serum albumin, the membrane was incubated overnight at 4°C with an appropriate dilution of the primary antibody [1∶2000 anti-E-cadherin mAb (clone 36/E-cadherin, BD), 1∶500 anti-occludin mAbs (clone 19/occludin, BD), 1∶2000 anti-vimentin mAbs (clone RV202, BD), 1∶1000 anti-fibronectin mAbs (10/fibronectin, BD), 1∶5000 anti-GAPDH polyclonal antibody (Santa Cruz Biotechnology), 1∶2000 anti-smad3 mAb (Cell Signaling Technology, Danvers, MA, USA), 1∶2000 anti-phospho-smad3 mAb (Cell Signaling Technology), 1∶2000 anti-smad2 mAb (Cell Signaling Technology), 1∶2000 anti-phospho-smad2 mAb (Cell Signaling Technology), 1∶2000 anti-ERK1/2 mAb (Cell Signaling Technology) and 1∶2000 anti-phospho-ERK1/2 mAb (Cell Signaling Technology)]. After washing, the membrane was incubated for 1 hr at room temperature with secondary antibody [horseradish peroxidase-conjugated sheep anti-mouse immunoglobulin (GE Healthcare, Piscataway, NJ) or horseradish peroxidase-conjugated donkey anti-rabbit immunoglobulin (GE Healthcare)]. The antibody complexes were visualized using the Amersham ECL detection system (GE Healthcare) as directed by the manufacturer.

### Transfection

Transfections were performed using Lipofectamine 2000 (Invitrogen) according to the manufacturer's instructions. The CXCL12-siRNA and CXCL3-siRNA were purchased from Sigma-Aldrich Japan (Ishikari Hokkaido). The sequences of siRNA are as follows.

CCL3: 5′-AAGCGAAGCCGGCAGGUCUTT-3′ (sense)

  5′-AGACCUGCCGGCUUCGCUUTT-3′ (antisense)

CXCL12:  5′-CAUUAUUGUACUUGCCUUATT-3′ (sense)

  5′-UAAGGCAAGUACAAUAAU-3′ (antisense)

### TGF-β_1_ ELISA

TGF-β_1_ ELISA kit specific for human TGF-β_1_ (TGF-β_1_ ELISA, IBL, Flughafenstrasse, Hamburg) was used according to the manufacturer's protocol. Briefly, 100 µl of samples was added to the TGF-β_1_ mAb pre-coated plate and incubated overnight at 4°C. After washing, 100 µl of antiserum was added and incubated for 2 hours at room temperature for blocking. After washing, anti-mouse IgG antibody conjugated to biotin was added and incubated for 45 minutes at room temperature. The color was developed with 100 µl of streptavidin peroxidase and substrate.

### Cell migration and invasion assay

Cytoselect 24-well cell migration assay kit (Cell Biolabs, Inc., San Diego, CA) and Cytoselect 24-well cell invasion assay kit (Cell Biolabs, Inc.) were used according to the manufacturer's protocol. Briefly, 500 µl of RPMI 1640 supplemented with 10% FBS was added to each well of the plate. Then, 300 µl of cell suspension containing 3×10^5^ cells/ml or 1.5×10^6^ cells/ml was added to the 8 µm pore size polycarbonate membrane cell culture inserts for cell migration assay or to the 8 µm pore size polycarbonate membrane cell culture inserts coated with a uniform layer of basement membrane matrix solution for cell invasion assay, respectively. Then, cells were incubated for 24 hrs at 37°C in a 5% CO_2_ atmosphere. Next, the medium was removed from the inside of the insert and the interior of the insert was gently swabbed with the wet end of cotton-tipped swabs to remove non-migratory cells. Then, each insert was transferred to a clean well containing 400 µl of cell stain solution and incubated for 10 minutes at room temperature. After washing, inserts were transferred to an empty well and 200 µl of extraction solution was added and incubated for 10 minutes. Then, the optical density (OD) was measured at 560 nm.

### Transwell co-culture system

Cytoselect 24-well cell migration assay kit (Cell Biolabs, Inc.) was used. HK cells without TGF-β_1_ treatment with/without PBMCs were co-cultured in the lower well with complete media and HK-2 cells pre-stimulated with TGF-β_1_ (10.0, 1.0 and 0.1 ng/ml) for 24 hrs followed by three careful washes by PBS or HK-2 cells without TGF-β_1_ stimulation were added to 2 µm pore size polycarbonate membrane cell culture inserts. Then, after 24 hrs of incubation, TGF-β_1_ concentration in the lower well media was measured by TGF-β_1_ ELISA.

### Statistical analysis

Statistical significance was evaluated by Student's t-test or by one-way analysis of variance followed by Fisher's PLSD test. Statistical significance was defined as P<0.05. All values are expressed as means ± SE.

## Supporting Information

Figure S1 **The change of CCL3 and CXCL12 expression in CCL3 and CXCL12 knockdown HK-2 cells by TGF-β_1_ stimulation.** CCL3 knockdown HK-2 cells (CCL3 KD) and CXCL12 knockdown HK-2 cells (CXCL 12KD) were induced by transfection of CCL3 siRNA or CXCL12 siRNA, respectively. Then, CCL3 KD and CXCL12 KD HK-2 cells were stimulated with TGF-β_1_ (10.0, 1.0 and 0.1 ng/ml) for 24 hrs. Non-treated HK-2 cells (Mock) and non-targeted-siRNA-transfected HK-2 cells (Control) served as controls. The expressions of CCL3 and CXCL12 were analyzed by real-time RT-PCR. CCL3 was up-regulated for CXCL12 KD, Mock and Control; however, it was not up-regulated for CCL3KD (upper graph) regardless of TGF-β_1_ stimulation. CXCL12 was up-regulated for CCL3 KD, Mock and Control; however, it was not up-regulated for CXCL12 KD (lower graph). Values are expressed as mean ± SEM of at least three experiments. *P<0.05.
(TIF)

Figure S2 **Effects of CCL3 and CXCL12 of HK-2 cells on the change of EMT markers on HK-2 cells by PBMCs.** PBMCs were co-cultured with non-treated HK-2 cells (Mock), CXCL12 knockdown HK-2 cells (CXCL12 KD), CCL3 knockdown HK-2 cells (CCL3 KD) or non-targeted-siRNA-transfected HK-2 cells (Control) for 24 hrs following TGF-β_1_ (0.1 ng/ml) stimulation for 24 hrs. Then, the changes of the epithelial markers (E-cadherin and occludin) and the mesenchymal markers (vimentin and fibronectin 1) on those HK-2 cells were assessed by real-time RT-PCR. The expression of epithelial cell junction markers (E-cadherin and occludin) decreased and that of mesenchymal markers (vimentin and fibronectin 1) increased in Mock, CCL3 KD and Control, whereas these changes were significantly decreased in CXCL12 KD. Values are expressed as mean ± SEM of at least three experiments. *P<0.05.
(TIF)

Figure S3 **Assessment of the effect of CCL3 and CXCL12 of HK-2 cells for morphology and EMT marker change by PBMCs.** PBMCs were co-cultured with non-treated HK-2 cells (Mock), CXCL12 knockdown HK-2 cells (CXCL12 KD), CCL3 knockdown HK-2 cells (CCL3 KD) or non-targeted-siRNA-transfected HK-2 cells (Control ) for 24 hrs following TGF-β_1_ (0.1 ng/ml) stimulation for 24 hrs. The cells were fluorescence-labeled by CM-Dil. Then, the cell morphology was observed using a fluorescent microscope. Magnification 100×. The phase contrast microscopy analysis showed that CXCL12 KD (C,D) changed less, morphologically, from a cobblestone-like monolayer to spindle-shaped fibroblast-like cells, than Mock (A,B), CCL3KD (E, F) and Control (G,H). Western blot analysis showed that the expression of epithelial cell junction markers (E-cadherin and occludin) decreased and that of mesenchymal markers (vimentin and fibronectin) increased in mock, CCL3KD and Control HK-2 cells, whereas these changes significantly decreased in CXCL12KD HK-2 cells (I). Values are expressed as mean ± SEM of at least three experiments. *P<0.05.
(TIF)
