# Supplementary figures and images for: The Interaction of LFA-1 on Mononuclear Cells and ICAM-1 on Tubular Epithelial Cells Accelerates TGF-β1-Induced Renal Epithelial-Mesenchymal Transition
Source: PLoS One. 2011 Aug 5;6(8):e23267. doi: 10.1371/journal.pone.0023267 (PMC3151298; doi:10.1371/journal.pone.0023267)

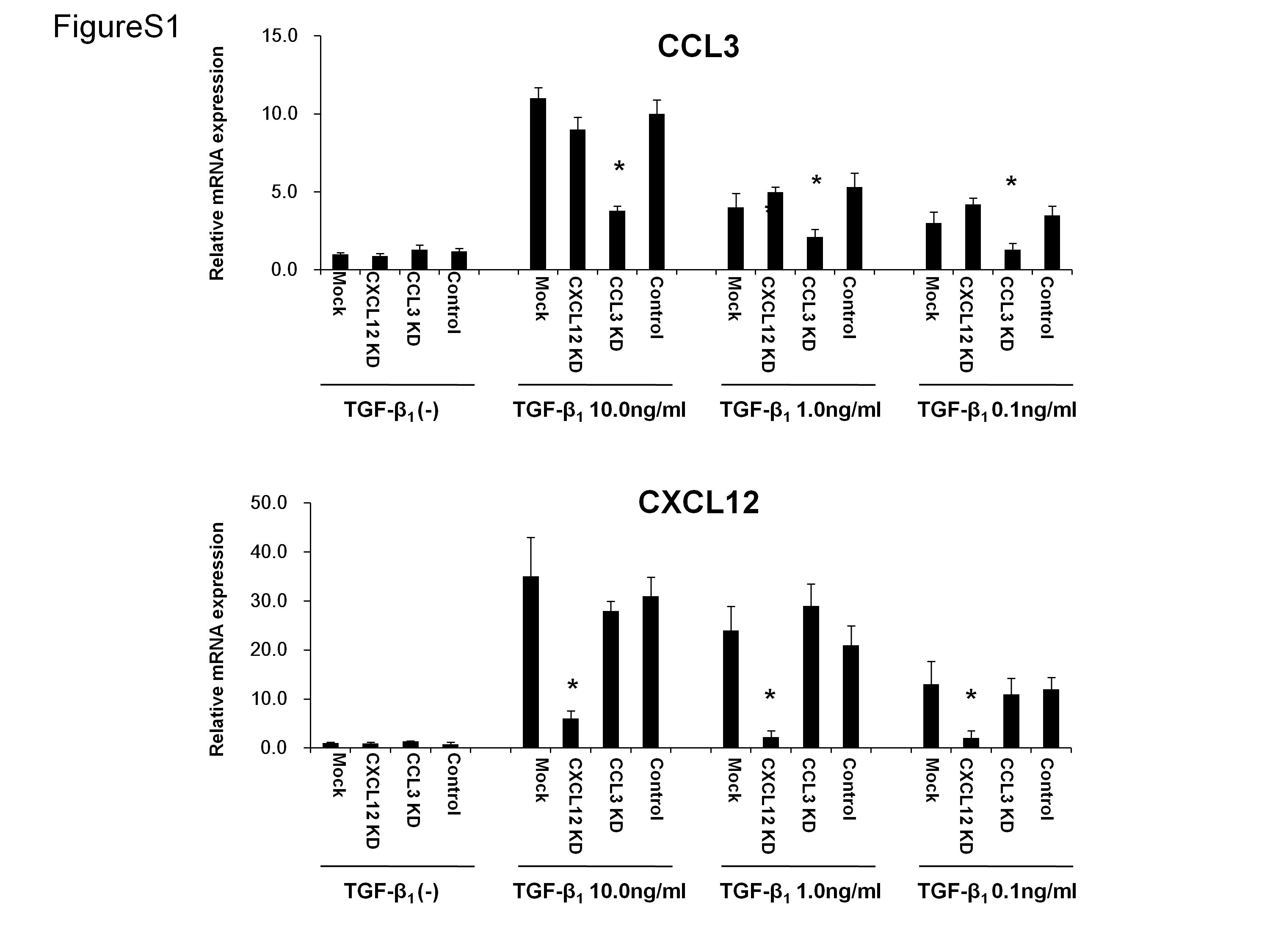

Supplement: Figure S1 — The change of CCL3 and CXCL12 expression in CCL3 and CXCL12 knockdown HK-2 cells by TGF-β1 stimulation. CCL3 knockdown HK-2 cells (CCL3 KD) and CXCL12 knockdown HK-2 cells (CXCL 12KD) were induced by transfection of CCL3 siRNA or CXCL12 siRNA, respectively. Then, CCL3 KD and CXCL12 KD HK-2 cells were stimulated with TGF-β1 (10.0, 1.0 and 0.1 ng/ml) for 24 hrs. Non-treated HK-2 cells (Mock) and non-targeted-siRNA-transfected HK-2 cells (Control) served as controls. The expressions of CCL3 and CXCL12 were analyzed by real-time RT-PCR. CCL3 was up-regulated for CXCL12 KD, Mock and Control; however, it was not up-regulated for CCL3KD (upper graph) regardless of TGF-β1 stimulation. CXCL12 was up-regulated for CCL3 KD, Mock and Control; however, it was not up-regulated for CXCL12 KD (lower graph). Values are expressed as mean ± SEM of at least three experiments. *P<0.05. (TIF) [file pone.0023267.s001.tif]

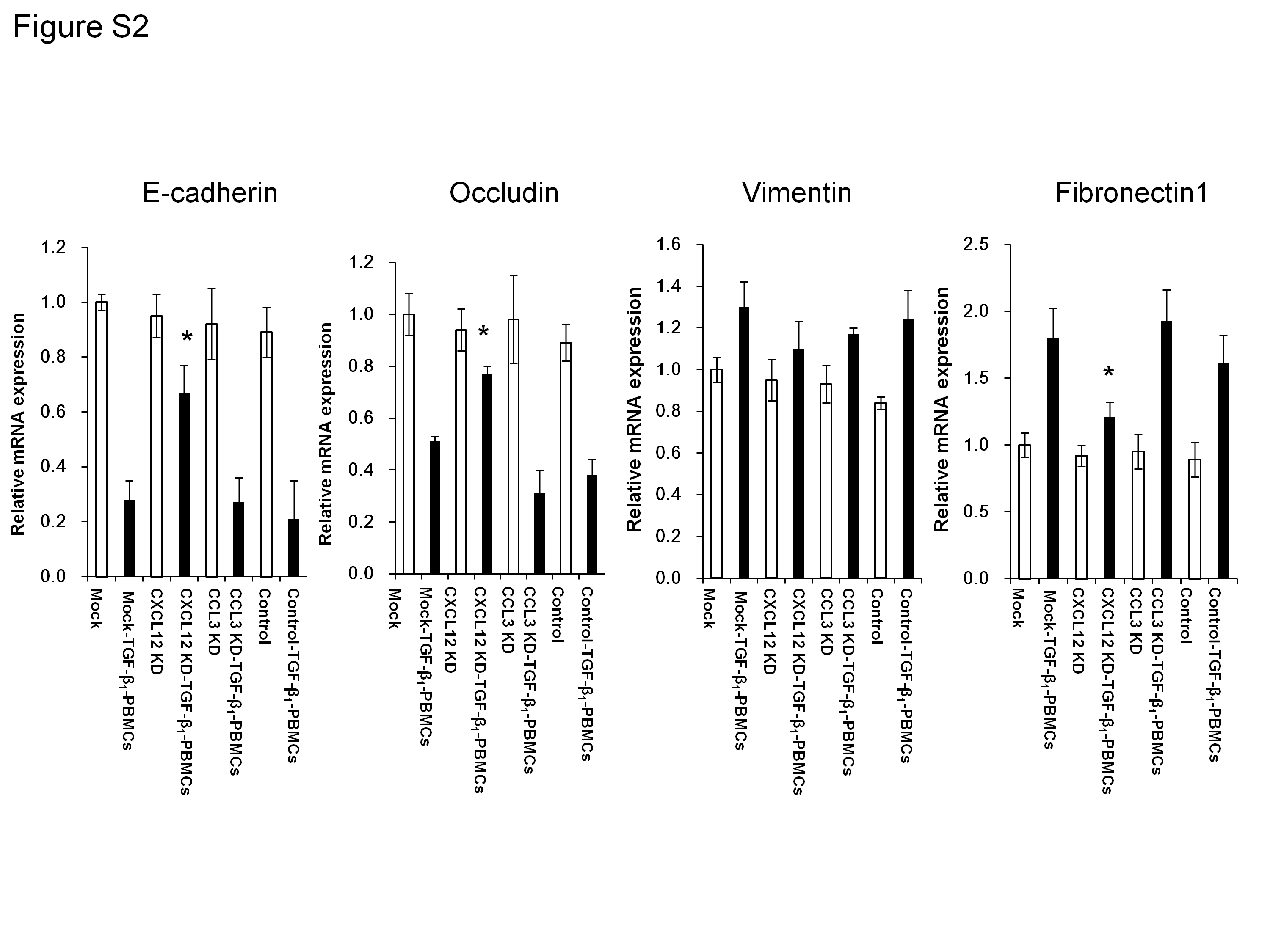

Supplement: Figure S2 — Effects of CCL3 and CXCL12 of HK-2 cells on the change of EMT markers on HK-2 cells by PBMCs. PBMCs were co-cultured with non-treated HK-2 cells (Mock), CXCL12 knockdown HK-2 cells (CXCL12 KD), CCL3 knockdown HK-2 cells (CCL3 KD) or non-targeted-siRNA-transfected HK-2 cells (Control) for 24 hrs following TGF-β1 (0.1 ng/ml) stimulation for 24 hrs. Then, the changes of the epithelial markers (E-cadherin and occludin) and the mesenchymal markers (vimentin and fibronectin 1) on those HK-2 cells were assessed by real-time RT-PCR. The expression of epithelial cell junction markers (E-cadherin and occludin) decreased and that of mesenchymal markers (vimentin and fibronectin 1) increased in Mock, CCL3 KD and Control, whereas these changes were significantly decreased in CXCL12 KD. Values are expressed as mean ± SEM of at least three experiments. *P<0.05. (TIF) [file pone.0023267.s002.tif]

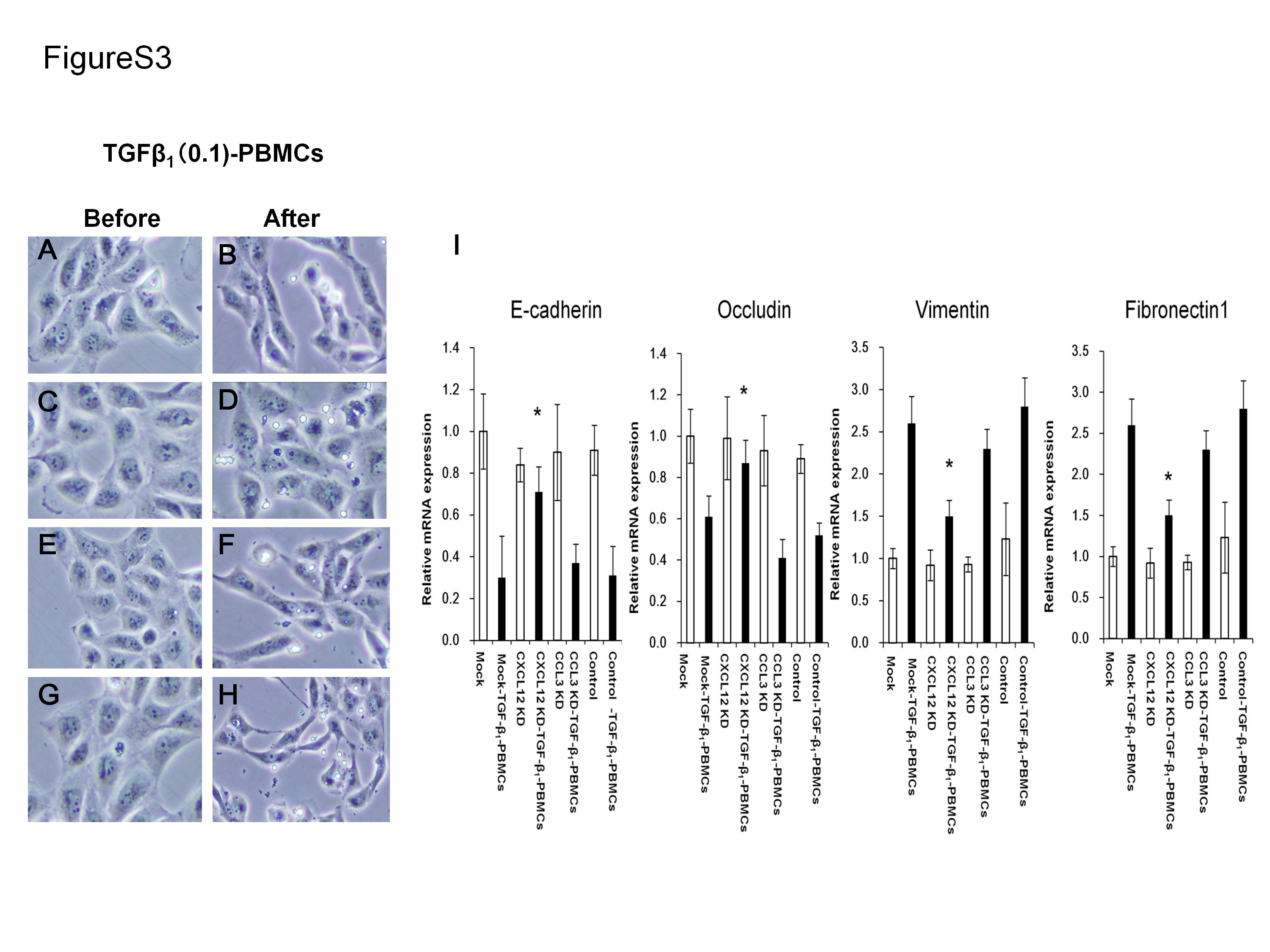

Supplement: Figure S3 — Assessment of the effect of CCL3 and CXCL12 of HK-2 cells for morphology and EMT marker change by PBMCs. PBMCs were co-cultured with non-treated HK-2 cells (Mock), CXCL12 knockdown HK-2 cells (CXCL12 KD), CCL3 knockdown HK-2 cells (CCL3 KD) or non-targeted-siRNA-transfected HK-2 cells (Control ) for 24 hrs following TGF-β1 (0.1 ng/ml) stimulation for 24 hrs. The cells were fluorescence-labeled by CM-Dil. Then, the cell morphology was observed using a fluorescent microscope. Magnification 100×. The phase contrast microscopy analysis showed that CXCL12 KD (C,D) changed less, morphologically, from a cobblestone-like monolayer to spindle-shaped fibroblast-like cells, than Mock (A,B), CCL3KD (E, F) and Control (G,H). Western blot analysis showed that the expression of epithelial cell junction markers (E-cadherin and occludin) decreased and that of mesenchymal markers (vimentin and fibronectin) increased in mock, CCL3KD and Control HK-2 cells, whereas these changes significantly decreased in CXCL12KD HK-2 cells (I). Values are expressed as mean ± SEM of at least three experiments. *P<0.05. (TIF) [file pone.0023267.s003.tif]
